# Block copolymer self-assembly derived mesoporous magnetic materials with three-dimensionally (3D) co-continuous gyroid nanostructure†

**DOI:** 10.1039/d3sm01622f

**Published:** 2024-03-05

**Authors:** Amaury Jousset Drouhin, William R. T. Tait, William Moore, Fei Yu, Yuanzhi Li, Jörg G. Werner, R. Bruce van Dover, Ulrich B. Wiesner

**Affiliations:** a Department of Materials Science and Engineering, Cornell University Ithaca USA ubw1@cornell.edu; b Department of Chemistry and Chemical Biology, Cornell University Ithaca USA; c Robert Frederick Smith School of Chemical and Biomolecular Engineering, Cornell University Ithaca USA; d Department of Mechanical Engineering, Boston University Boston USA; e Division of Materials Science and Engineering, Boston University Boston USA

## Abstract

Magnetic nanomaterials are gaining interest for their many applications in technological areas from information science and computing to next-generation quantum energy materials. While magnetic materials have historically been nanostructured through techniques such as lithography and molecular beam epitaxy, there has recently been growing interest in using soft matter self-assembly. In this work, a triblock terpolymer, poly(isoprene-*block*-styrene-*block*-ethylene oxide) (ISO), is used as a structure directing agent for aluminosilicate sol nanoparticles and magnetic material precursors to generate organic–inorganic bulk hybrid films with co-continuous morphology. After thermal processing into mesoporous materials, results from a combination of small angle X-ray scattering (SAXS) and scanning electron microscopy (SEM) are consistent with the double gyroid morphology. Nitrogen sorption measurements reveal a type IV isotherm with H1 hysteresis, and yield a specific surface area of around 200 m^2^ g^−1^ and an average pore size of 23 nm. The magnetization of the mesostructured material as a function of applied field shows magnetic hysteresis and coercivity at 300 K and 10 K. Comparison of magnetic measurements between the mesoporous gyroid and an unstructured bulk magnetic material, derived from the identical inorganic precursors, reveals the structured material exhibits a coercivity of 250 Oe, opposed to 148 Oe for the unstructured at 10 K, and presence of remnant magnetic moment not conventionally found in bulk hematite; both of these properties are attributed to the mesostructure. This scalable route to mesoporous magnetic materials with co-continuous morphologies from block copolymer self-assembly may provide a pathway to advanced magnetic nanomaterials with a range of potential applications.

## Abstract 2

This work elucidates a route to mesoporous magnetic materials with co-continuous morphologies from block copolymer self-assembly. The co-continuous structure impacts the magnetic behavior compared to non-structured chemically-identical materials.

## Graphical abstract

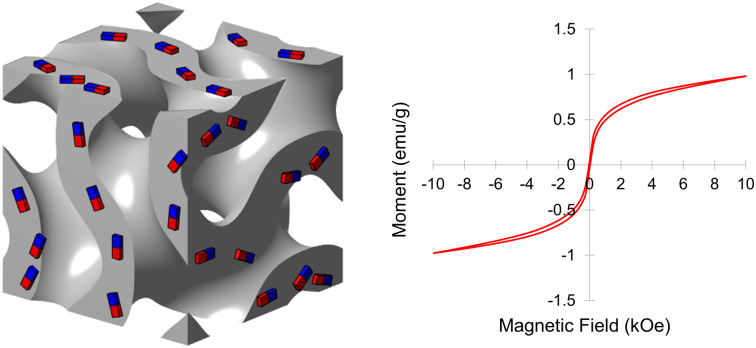

## 1. Introduction

Nanostructured magnetic materials and nanomagnet arrays have recently garnered increasing interest due to their potential in myriad applications including magnetic cooling, logic gates, memory devices, quantum computing, nanomedicine, catalysis, and spintronics.^1–4^ Many of these applications are appealing as they are at the frontier of the ever-growing field of quantum materials, offering much fertile ground in both basic scientific understanding and technological development. Control over the size, shape, and arrangement of nanomagnetic materials is crucial for achieving desired properties and performance for a given application. To this end, numerous techniques have been developed to create ordered arrays of nanomagnetic materials. Although conventional methods, including lithography, focused ion beams, and molecular beam epitaxy,^5–7^ have demonstrated successful fabrication of nanomagnetic arrays, researchers have long sought more efficient and cost-effective alternatives.

While for the most part the fields of soft matter and hard condensed matter have developed apart from each other, recent efforts in combining these two fields have yielded success in generating advanced nanomagnetic materials. Compared to conventional methodologies, fabrication techniques with soft matter offer the use of solution-processible, self-assembling materials to template or structure-direct hard magnetic materials into ordered structures. Using soft matter as a way to structure magnetic materials should open doors to new research of fundamental and technological relevance by offering morphological control at the mesoscale, allowing the use of a wide variety of materials classes, and providing access to scalable solution-based synthesis approaches to a range of form factors.^8^ Several soft matter methodologies of generating nanomagnetic arrays have already been investigated, such as using liquid crystal templating,^9,10^ colloidal assembly,^11,12^ micellar templating,^13^ surfactant templating,^14,15^ and block copolymer (BCP) self-assembly (SA) based templating and structure direction.^15–18^ Of particular interest to the authors are BCPs. This class of soft matter consists of two or more covalently bound, chemically distinct polymer chains, or “blocks.” These unalike blocks will undergo segregation at the mesoscale, depending on the Flory–Huggins monomer–monomer interaction parameter and block volume fraction; this leads to BCP SA into a variety of mesoscopic morphologies, including micellar structures, hexagonally packed cylinders, lamellae, and co-continuous network structures.^19–21^ Understanding the phase behavior of a BCP allows for a tailored synthesis to target a specific morphology. Furthermore, assuming enthalpic and entropic requirements are met, blocks can be selectively swelled with additives in order to increase their respective volume fractions and tune morphology.^22^ These additives themselves can be functional materials, such as sol–gel derived silicate^23,24^ or transition metal oxide^25,26^ nanoparticles, effectively using BCPs as structure directing agents to co-assemble hybrids with morphologies informed by the parent BCP phase behavior.^27^

Previous work using BCPs has explored soft templating of nanomagnetic hexagonal arrays through *in-situ* sol–gel routes, or deposition of magnetic nickel to pre-self-assembled polymers.^16–18,28^ However, these works have either been limited in the complexity of their formed morphologies or required multiple steps, such as electrodeposition or electroless deposition into a self-assembled soft template. In this work we report a SA one-pot synthesis using a sol–gel method, and subsequent thermal processing, to generate a mesoporous inorganic material consistent with co-continuous gyroidal morphology, with weak ferromagnetic (WF) response at 300 K and ferromagnetic hysteresis behavior at 10 K, using a triblock terpolymer, poly(isoprene-*block*-styrene-*block*-ethylene oxide) (PI-*b*-PS-*b*-PEO, or simply ISO), as a structure-directing agent. To that end an aluminosilicate-metal oxide-precursor hybrid was first synthesized using iron(iii) ethoxide as an iron oxide precursor and then converted *via* high-temperature treatment into a mesoporous nanostructure with magnetic properties.^16^ The development of the resulting magnetic nanomaterials is in the hope of expanding the library of co-continuous, nanostructured magnetic materials that can benefit from high surface area and processing techniques to expand and improve applications of such materials. The impact of such materials is expected to range from green catalysts to quantum materials.

## 2. Materials and methods

### 2.1. Materials

1,1-Diphenylethylene (DPE) (97%, Aldrich), *n*-butyllithium (1.6 M in hexanes, Sigma-Aldrich), sec-butyllithium (1.4 M in cyclohexane, Sigma-Aldrich), di-*n*-butylmagnesium (1 M in ether and hexanes, Sigma-Aldrich), methanolic hydrogen chloride (methanolic HCl)(3 N, Sigma-Aldrich), hydrochloric acid (HCl) (38%, EMD Millipore Corp.), 3-(glycidyloxypropyl) trimethoxysilane (>98%, Aldrich), aluminum tri-sec butoxide (97%, Acros Organics), potassium chloride (KCl) (99.95%, Alfa Aesar), iron(iii) ethoxide (Chem Cruz), and cobalt chloride (anhydrous 99.8%, Chem-Impex International, Inc.) were used as received. Tetrahydrofuran (THF) (99.9% anhydrous, Sigma-Aldrich) was used after distillation over *n*-butyllithium and DPE for polymerization reactions, and after being dried over molecular sieves for the formation of final polymer solutions. Benzene (99.8% anhydrous, Sigma-Aldrich) was used after distillation over *n*-butyllithium and DPE for synthesis. Chloroform (Sigma-Aldrich) was used after drying over molecular sieves for the formation of polymer solutions. Isoprene (99%, contains <1000 ppm *p-tert*-butylcatechol as inhibitor, Sigma-Aldrich) was used after distillation of *n*-butyllithium, styrene (>99%, contains 4-*tert*-butylcatechol as stabilizer, Sigma-Aldrich) was used after distillation over di-*n*-butylmagnesium, and ethylene oxide (99.5% Sigma-Aldrich) was used after distillation over *n*-butyllithium for the BCP synthesis. Potassium naphthalimide was produced by mixing potassium, naphthlene, and THF.

### 2.2. Methods

#### 2.2.1. ISO polymer synthesis

ISO was synthesized *via* sequential anionic polymerization as described in detail elsewhere.^22,29^ Briefly, a desired amount of sec-butyllithium was added to a reactor of clean benzene after it was pumped into a nitrogen glove box. Isoprene was added to the reactor and allowed to react overnight. An aliquot was taken and quenched with methanolic HCl for GPC analysis. Next, styrene was added to the reactor and reacted overnight. The following day, a PI-*b*-PS aliquot was taken and quenched with methanolic HCl for GPC analysis. The reactor was removed from the glove box and fitted with a reverse injection apparatus. Ethylene oxide was distilled over *n*-butyllithium, and then reverse injected into the reactor. The resulting PI-*b*-PS end-capped with one unit of ethylene oxide (ISOH) was subsequentially quenched with methanolic HCl.

For addition of the third block, the ISOH was neutralized with sodium bicarbonate solution. Subsequently, the organic layer was washed with deionized water. The organic layer of the separation was then collected, and the ISOH was concentrated down and subsequently freeze-dried over a period of several days before being redissolved in THF. Potassium naphthalimide was used to reinitiate the ISOH polymer, by titrating it into the reactor until there was a consistent light green color. Then, a desired amount of ethylene oxide (EO) was reverse injected into the reactor. The reactor was left stirring at 40 °C for four days, followed by a quench with methanolic HCl. The resultant terpolymer was then neutralized and dried prior to use. Of note, EO is a toxic, flammable gas and should be handled and stored properly according to safety data sheet guidelines.

#### 2.2.2. Aluminosilicate sol preparation

The aluminosilicate sol was prepared through a hydrolytic route.^24^ First, 2.65 g of 3-(glycidyloxypropyl)trimethoxysilane (GLYMO) was added to a 20 mL vial through a 0.2 μm syringe filter followed by 0.7 g of aluminum tri-sec butoxide (resulting in a molar ratio of 8 : 2 GLYMO: aluminum tri-sec butoxide) and 20 mg of KCl. The solution was then stirred in an ice-water bath for 10 minutes and prepared using a two-step acid catalyzed hydrolysis procedure. 0.135 mL of 0.1 M HCl was added dropwise (1 drop every 3 seconds) through a 21 G needle. Once added, the solution was stirred 15 minutes at 0 °C, followed by 15 minutes of stirring at 21 °C. 0.85 mL of 0.1 M HCl was then added dropwise through a 22 G needle (1 drop every 7 seconds) to the resulting cloudy solution, followed by 20 minutes of stirring at 21 °C. The now clear to semi-clear solution was filtered through a 0.2 μm syringe filter.

#### 2.2.3. Hybrid preparation

Seventy-five milligrams of ISO were dissolved to a 2 wt% solution into a 1 : 1 mass ratio of anhydrous tetrahydrofuran and anhydrous chloroform. Iron(iii) ethoxide was then added to the polymer solution. An appropriate weight of aluminosilicate solution (assuming 53 wt% inorganic content) was added to the polymer solution in a 75 : 25 molar ratio of aluminosilicate precursors (excluding HCl) and iron(iii) ethoxide, to reach a 62% weight fraction PEO + inorganic to polymer. Assuming full conversion of the inorganic precursors to in-framework aluminosilicate network and iron(iii) oxide (*i.e.*, hematite, α-Fe_2_O_3_, see main text below), this translates to a weight fraction of about 30% hematite converted to a volume fraction of about 10% of hematite (assuming a density of 1.4 g cm^−3^ for the aluminosilicate^23^ and 5.26 g cm^−3^ for hematite) for the fully converted mesoporous inorganic material. The solution was then stirred for 1 hour, and casted onto polytetrafluoroethylene (PTFE) cups under a glass dome, all on a hot plate set to 50 °C overnight. The resulting films were then heated in a vacuum oven for 1 hour at 130 °C and then removed to cool to room temperature.

#### 2.2.4. Porous inorganic preparation

The hybrid films were calcined to promote nucleation and growth of the oxides in parallel to the thermal decomposition of the terpolymer. To that end, the hybrids were calcined at 350 °C for 3 h followed by 550 °C for 6 hours at a ramp rate of 1 °C min^−1^ for both steps. The slow ramp rate facilitated thermal decomposition of the polymer while maintaining the mesostructure.

#### 2.2.5. Characterization

##### ISO triblock terpolymer

Terpolymer characterization was performed *via* a combination of gel permeation chromatography (GPC) and proton nuclear magnetic resonance (^1^H-NMR). GPC was run on an Agilent Series 1100 LC system with Series 1200 Quaternary Pump and Degasser, equipped with an Agilent ResiPore GPC column. The system was kept at 30 °C and 14 bar. Data was taken with an RI detector, with samples run dissolved in THF at concentrations between 0.5 and 1 mg mL^−1^. NMR was run on a Bruker av500 NMR spectrometer at 500 MHz equipped with a liquid nitrogen-cooled cryoprobe; the samples were dissolved in deuterated chloroform.

##### Hybrid materials

Small angle X-ray scattering (SAXS) was conducted to elucidate the morphology of the hybrid materials. SAXS was run at the cornell high energy synchrotron source (CHESS) BioSAXS line; the X-ray wavelength used was 1.0938 Angstrom (Å), the energy was 11.3 keV, and the sample-to-detector distance was 1776 mm. SAXS patterns were analyzed on IgorPro 9.00.^30^ Scanning electron microscopy (SEM) was conducted on a Zeiss Gemini 500 scanning electron microscope with a secondary electron detector at a 3 kV accelerating voltage. Samples were sputter-coated with gold-palladium before imaging. Nitrogen sorption was conducted on an ASAP 2020 surface area and porosity analyzer. The analysis took place at −196 °C after the sample was degassed. X-ray diffraction (XRD) was run on a Bruker D8 advance ECO powder diffractometer with Cu-Kα source. The scan speed used for XRD was 2° min^−1^. Sherrer analysis of the diffractogram was done using MDI JADE software with Pseudo-Voigt peak fitting on the (104) and (110) peaks on the diffractogram, taking the average of the two resultant values. Magnetometry measurements were performed on a quantum design MPMS3 superconducting quantum interference device (SQUID) vibrating-sample magnetometer (VSM). The magnetization *versus* field measurements were taken at 300 kelvin (K) and 10 K between ±10 000 Oersted (Oe). The zero-field cooling and field cooling measurements were taken between 2–300 K, with an applied field of 100 Oe during the field cooling measurement.

## 3. Results and discussion

**Fig. 1:**
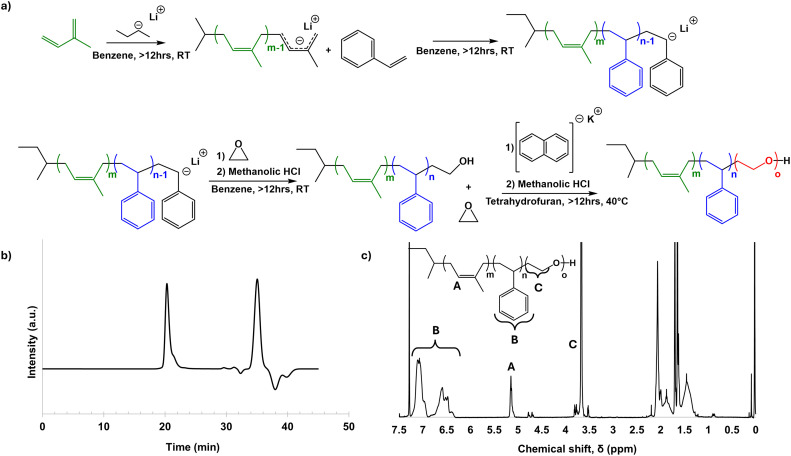
Schematic of ISO synthesis (a), and GPC (b), and proton NMR (c) characterization of the final ISO terpolymer.

ISO was successfully synthesized by sequential anionic polymerization (Fig. 1a). By GPC, the ISO was found to have a dispersity *Đ* of 1.14. The peak centered at 20.3 minutes was used to determine *Đ*, and all other signals in the elugram past 25 minutes were considered unreacted monomer or other small molecules generated when the living polymer was quenched (Fig. 1b). GPC of the poly(isoprene) (PI) and poly(isoprene-*block*-styrene) (PI-*b*-PS) diblock copolymer were also taken (Fig. S1a and b, ESI†), which were used to help determine the final molar mass in conjunction with NMR, as well as track molar mass and *Đ* throughout the synthesis process (Table S1, ESI†). Proton NMR was used to determine the molar ratio, and subsequently the mass and volume ratios, of the final ISO terpolymer. Peaks in ^1^H-NMR associated with unique protons in each block were integrated to determine the monomer ratio (Fig. 1c). For the poly(isoprene) (PI) block, peaks from 5.0–5.2 ppm and 4.6–4.8 ppm were integrated, representing the single proton from 1,4-PI and the two protons from 1,2-PI and 3,4-PI, respectively. The PI in this ISO was predominantly 1,4-polyisoprene. The poly(styrene) (PS) block was identified by the signal from 6.25–7.25 ppm, corresponding to the 5 protons of the phenyl ring of the polymer. The poly(ethylene oxide) (PEO) block was identified by peaks from 3.5–3.9 ppm, corresponding to the four protons on the carbons in the repeat unit of the polymer. Integrating these peaks, and then dividing each respective intensity by the number of associated protons yielded a monomer ratio, which was then converted to a mass ratio. The ISO synthesized had a 14 : 36.4 : 49.6 PI : PS : PEO weight ratio. From the mass determined for the PI block by GPC (Fig. S1a, ESI†) and the mass ratio determined by NMR, the ISO number average molar mass, *M*_n_, was determined to be 84 900 g mol^−1^.

**Fig. 2:**
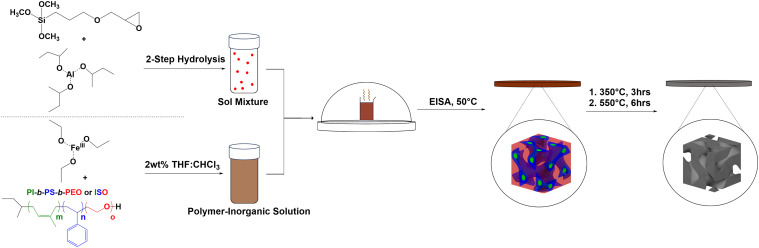
Schematic showing co-assembly of ISO and inorganic components. The aluminosilicate sol is prepared separately and then added into a solution of ISO terpolymer and iron oxide precursor. BCP SA directed nanostructured hybrid formation is then accomplished *via* EISA. Resulting self-assembled hybrid materials are calcined to produce the final mesoporous materials.

With the ISO fully characterized, a particularly desired morphology for the terpolymer/inorganic hybrid material could then be targeted. Since the PEO of ISO is the only hydrophilic block of the terpolymer, it is expected to be selectively swollen with hydrophilic sol additives, thus increasing the volume fraction of the “PEO + inorganic” block. A schematic of the synthetic approach is provided in Fig. 2. For the hybrid synthesis, a 2 wt% solution of ISO in 1 : 1 THF:CHCl_3_ by mass and a hydrolytic sol of aluminosilicate nanoparticles were separately prepared. Prior to mixing these two solutions, iron(iii) ethoxide was added to the polymer solution; the amount of iron(iii) ethoxide was chosen so that there would be a molar ratio of 75 : 25 aluminosilicate precursors to iron(iii) ethoxide. This ratio of aluminosilicate precursors to iron(iii) ethoxide allowed the block copolymer-imposed mesostructure to remain intact after the subsequent calcination-induced growth of the iron oxide crystallites. Indeed, synthesized hybrids with 50 : 50 aluminosilicate precursors to iron(iii) ethoxide showed crystal overgrowth and resulting loss of mesostructure retention upon calcination (Fig. S2, ESI†). After mixing these solutions in the appropriate ratios, the resultant dope was cast and allowed to assemble under evaporation induced SA (EISA). The resulting hybrid films were then calcined to yield monoliths of porous aluminosilicate with inorganic ferrous oxides expected to form in the pore walls of the aluminosilicate matrix. While sol–gel routes to iron oxides exist without a silicate matrix, the aluminosilicate chemistry used is known to be a well-behaved system for co-assembly with O-block containing BCPs.^23,24^ The aluminosilicate matrix also adds robustness and thermal stability to the material.^16^ Furthermore, since the aluminosilicate sol is synthesized prior to mixing with the BCP and iron oxide precursor, this material is thought to act as a confining agent resulting in discrete iron oxide nanoparticles distributed throughout the aluminosilicate matrix.

**Fig. 3:**
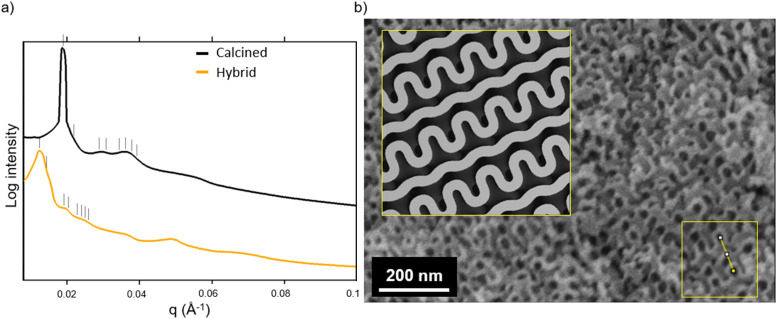
SAXS profiles of hybrids and thermally processed mesoporous materials (a). Ticks above each curve represent expected peak positions associated with a double gyroid structure. SEM of mesoporous material compared to a characteristic projection of a (211) plane of a double gyroid (b). The yellow line in the boxed region of the SEM consistent with the (211) plane of the double gyroid is 111 nm long.

**Fig. 4:**
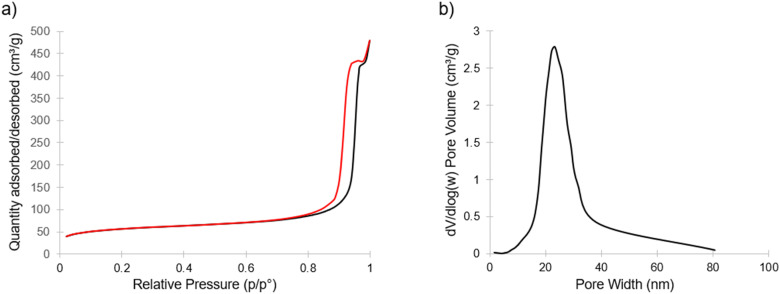
Nitrogen sorption isotherm of mesoporous material with adsorption (black) and desorption (red) branches (a). Resulting pore size distribution from the BJH model using the adsorption branch of the isotherm and centered around 23 nm (b).

Fig. 3a shows the SAXS traces of the organic–inorganic hybrid (orange, bottom) and the calcined inorganic material (black, top). Ticks above each trace indicate the first eight expected peak positions of the double gyroid with space group *Ia*3*d* (Q230) consistent with these patterns. From such SAXS results, however, unambiguous assignment of cubic co-continuous structures is very challenging as a result of significant peak broadening and a lack of well-defined higher order reflections. Fig. 3b shows a SEM cross-sectional image of the mesoporous material clearly exhibiting typical features expected from a co-continuous gyroid structure. This becomes evident from a comparison with a model-generated image of a characteristic double gyroid (211) plane shown in the inset of this figure panel. According to literature, the distance between the large wave-like features in the (211) plane of the double gyroid as shown by the yellow line in the yellow box in the lower right of the SEM image in Fig. 3b is equal to the unit cell size multiplied by 
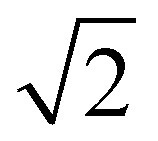
.^31^ The drawn line in the SEM of Fig. 4a is 111 nm long. Comparing the unit cell size of 78.5 nm ascertained from measuring the drawn line across the feature size of the (211) plane in the SEM image, to the *d*-spacing of 81.3 nm obtained from analysis of the SAXS pattern, we get reasonably good agreement between the two, consistent with the assignment of the structure to a double gyroid.

Fig. 4 shows nitrogen sorption/desorption results for the calcined porous material exhibiting type IV isotherms with H1 hysteresis, indicating an open pore structure.^32^ This hysteresis is consistent with what would be expected from the continuous open pore network suggested by SEM (Fig. 3b and Fig. S3, ESI†). From this data, the Brunauer–Emmett–Teller (BET) based data analysis yielded specific surface areas around 200 m^2^ g^−1^. The inset of Fig. 4 shows the Barrett–Joyner–Halenda (BJH) model derived pore size distribution obtained from the adsorption branch with an average pore size of approximately 23 nm. This pore size is in reasonable agreement with the pores visible in Fig. 3b.

**Fig. 5:**
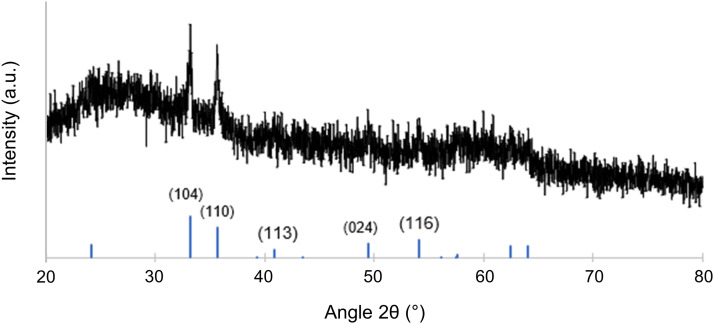
XRD of the calcined and mesoporous gyroidal aluminosilicate/Fe_2_O_3_ composite material with reference peaks for hematite (α-Fe_2_O_3_) shown at the bottom from PDF #01-087-1164.^35^

XRD measurement results on the mesoporous inorganic material are shown in Fig. 5. The aluminosilicate matrix is expected to be amorphous under the thermal processing conditions employed.^16^ Any XRD reflections are therefore expected to stem from crystalline iron oxide phases distributed in the porous aluminosilicate matrix. Indeed, on top of an amorphous halo likely from the aluminosilicate, the XRD results show peaks consistent with hematite (α-Fe_2_O_3_). Pseudo-Voigt fitting and subsequent Sherrer analysis of the peaks located at 2*θ* = 33.2° and 35.6° were averaged to yield a particle size of about 20 nm. Due to the resolution of the data, it is possible that some magnetic phases such as magnetite, or maghemite (γ-Fe_2_O_3_) exist in the material as minority components that are being overlooked. Fig. S4 (ESI†) shows XRD results of an aluminosilicate with iron oxide embedded in it synthesized without any BCP structure directing agent; this bulk material had a similar XRD pattern to Fig. 5, and it is shown with hematite and magnetite reference peaks. There are examples in literature of iron oxide nanoparticles embedded in inorganic matrices producing both γ-Fe_2_O_3_^16^ and α-Fe_2_O_3_ at or above the processing temperature used in this work.^33,34^ While the current signal-to-noise level from experiments on a powder XRD diffractometer, *i.e.*, in the absence of synchrotron data, does not allow to assess whether there are any other phases present in the material, the results shown in Fig. 5 clearly suggest α-Fe_2_O_3_ in an amorphous aluminosilicate matrix.

**Fig. 6:**
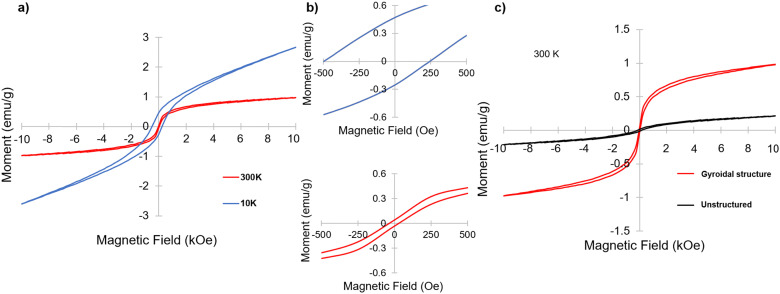
Magnetometry measurements of magnetic moment *vs*. applied magnetic field (*M–H*) at 300 K (red) and 10 K (blue) for the BCP-derived mesoporous gyroidal material (a). Zoomed in portions of curves in (a) to show the hysteresis at 0 applied field for each of the curves (b). Comparison of the *M*–*H* curves at 300 K of BCP-derived mesoporous gyroidal material (red) and bulk sample (black) obtained without use of ISO as structure directing agent (c).

Having identified α-Fe_2_O_3_ phase in the resulting mesoporous materials, we were interested in its magnetic properties, in particular under the nanoconfinement of the co-continuous gyroidal aluminosilicate mesostructure. To that end, magnetometry measurements were performed. Fig. 6a and b show two magnetization *versus* magnetic field (*M*–*H*) curves taken at a high and low temperature (300 K and 10 K, respectively); both curves demonstrate magnetic hysteresis. This hysteresis was particularly noticeable at 10 K, with remnant magnetization and coercivity of approximately 0.47 emu g^−1^ and 250 Oe, respectively, compared to 0.067 emu g^−1^ and 32 Oe, respectively, at 300 K. These results are indicative of weak ferromagnetism (WF) at 300 K and ferromagnetic hysteresis behavior at 10 K, which is consistent with results on other nanostructured hematite materials reported in the literature.^34,36,37^ Hematite nanoparticles are typically expected to be antiferromagnetic at low temperatures, and perfectly compensated antiferromagnets do not demonstrate hysteresis, so the results at 10 K indicate that the material is an antiferromagnet with uncompensated spins.^33^

To investigate the impact of the gyroidal nanostructure on the magnetic behavior of the aluminosilicate-templated α-Fe_2_O_3_, an inorganic sample was generated by the same procedure as the gyroid sample, but without using the ISO terpolymer as a structure directing agent. The resulting material was expected to still have iron oxide nanoparticles embedded in an aluminosilicate matrix, but, as a result of the lack of ISO, was expected to lack the mesoporous gyroid morphology imparted by the BCP. Fig. S4 (ESI†) shows the XRD of this sample, which is very similar to the one of the gyroidal material exhibited in Fig. 5, also consistent with predominantly α-Fe_2_O_3_ embedded in an amorphous aluminosilicate. Fig. 6c shows the comparison of the *M*–*H* curves at 300 K of the mesoporous inorganic structure-directed into a gyroidal morphology and the unstructured bulk inorganic. While both are magnetic, the inorganic material with gyroidal structure shows much stronger magnetization with field, as well as higher remnant magnetization at zero applied field. However, the coercivity of the unstructured sample is higher than that of the structured material at 300 K, with a value of approximately 147 Oe compared to 32 Oe, respectively. Comparing the *M*–*H* curves of the gyroidal and unstructured materials at 10 K, the coercivity and remnant magnetization of the structured inorganic is higher than that of the unstructured sample, with the unstructured sample having a coercivity of 148 Oe and a remnant magnetization of 0.034 emu g^−1^ (Fig. S5, ESI†). Furthermore, the change in coercivity from 300 K to 10 K in the unstructured inorganic was not large, increasing by only approximately 1 Oe, while the change in the coercivity of the structured material from 300 K to 10 K was significant, increasing from 32 to 250 Oe (Fig. 6a and Fig. S6, ESI†). Large coercivity and hysteresis has been observed in small hematite nanoparticles (5 nm) embedded in a porous silica matrix,^33^ but the origin of the hysteresis and coercivity of the gyroidal mesostructured material at 10 K is not explained by the aluminosilicate matrix here, since there was only a trivial change in coercivity of the unstructured material when *M*–*H* measurements were taken at 10 K.

Of note, the *M*–*H* curve taken of the gyroidal sample at 10 K showed asymmetric hysteresis about the origin (Fig. 6b, top panel). Asymmetric hysteresis and horizontal loop shifting in purely ferromagnetic materials has been recently described using micromagnetic simulations.^38^ The reason ascribed to the hysteresis asymmetry and shifting in these simulations was the interaction of intermixed weak and strong ferromagnets in the system caused by random orientation anisotropy in the different grains of the simulated nanoparticles.^38^ The hysteresis asymmetry and shifting observed in our materials may therefore be due to the orientation of the grains of magnetic nanoparticle embedded in the gyroidal aluminosilicate matrix. Of note, this behavior was not demonstrated by the unstructured sample. While this phenomenon needs to be explored more thoroughly, a magnetic material demonstrating pseudo-exchange bias behavior has implications for applications such as spintronics.

**Fig. 7:**
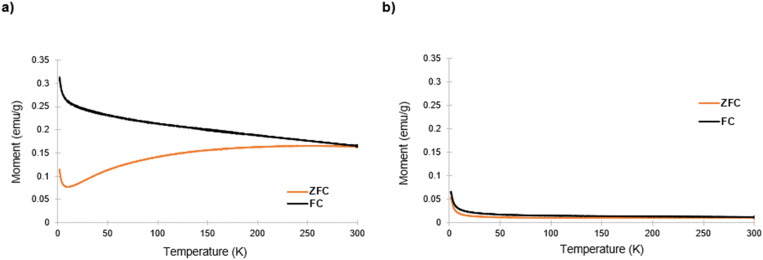
ZFC and FC curves for the BCP-derived mesoporous gyroidal aluminosilicate with imbedded α-Fe_2_O_3_ magnetic material (a) and the unstructured bulk aluminosilicate sample with imbedded α-Fe_2_O_3_ magnetic material (b).

Subsequently, zero-field (ZFC) and field cooling (FC) measurements were taken on both samples, with results shown in Fig. 7. At low temperatures, for the mesoporous gyroidal material, the ZFC and FC in Fig. 7a showed a significant separation, as is expected for ferromagnetic behavior. As the temperature increased to 300 K, the FC curve monotonically decreased while the ZFC curve, after an initial dip, monotonically increased, demonstrating an irreversibility temperature (*T*_irr_), where the two curves meet, >300 K. Literature explains the increase in the ZFC curve as surface spin ordering that can be increased by the large surface area to volume ratio of a porous structure.^34^ Of note, there was no indication of a clear blocking temperature in the ZFC curve after the initial decrease. Furthermore, bulk hematite is known to undergo a Morin transition at ∼265 K from weak ferromagnetic to antiferromagnetic behavior,^36^ usually displayed by a sharp peak in the ZFC and FC curves. Hematite nanoparticles also display Morin transitions, below which antiferromagnetic behavior is dominant, with the Morin transition temperature shifting to lower temperatures with decreasing particle size; below 10 nm particle size the Morin transition is suppressed.^34^ However, Tadic *et al.* found that hematite particles of even 40 nm size in a porous alumina matrix had a suppressed Morin transition when taking ZFC curves at 100 Oe.^34^Fig. 7a shows no indication of peaks corresponding to a Morin transition, indicating that it was suppressed. Sherrer analysis of the peaks in Fig. 5 suggested particles of about 20 nm diameter, indicating that these materials demonstrated a similar property as the materials described in Tadic *et al.*, *i.e.*, suppression of Morin transition in hematite nanoparticles with size above 10 nm. The shape of these curves is consistent with other hematite-based nanomaterials demonstrating magnetic hysteresis behavior.^34,36,37^

Fig. 7b shows the ZFC and FC curves for the unstructured bulk inorganic material, which display different behavior than those of the structured counterpart (zoomed-in *y*-axis shown in Fig. S7, ESI†). Both the ZFC and FC curve monotonically decrease with temperature to a much lower remnant magnetization value. Of note, the *T*_irr_ is still >300 K, and there is still no indication of a Morin transition in Fig. 7b. This implies that the suppression of the Morin transition is likely due to the confinement of the α-Fe_2_O_3_ phase in the aluminosilicate matrix and not to the gyroidal mesostructure. It is also possible that ZFC and FC measurements under high magnetic field may reveal the suppressed Morin transition temperature.^34^ Another feature of note is the lack of the local maximum in ZFC curve before divergence from the FC curve that indicates blocking temperature; blocking temperature indicates a switch to superparamagnetic ordering, which has been observed in some hematite nanomaterials with at least one dimension <10 nm.^33,34^ Lastly, it is clear that the mesoscale gyroidal structure impacts the values of the magnetic moment of the material when comparing Fig. 7a and b.

The results of the magnetic characterizations indicate that imposing the gyroidal mesostructure onto an aluminosilicate/α-Fe_2_O_3_ hybrid material does impact its magnetic behavior compared to a chemically identical material that is not mesostructured. There are several potential explanations for the phenomena observed. Above the Morin transition temperature, WF behavior in bulk hematite typically diminishes due to charge transfer from O 2p to Fe 3d, and strain and structural defects have been used to overcome this issue.^37^ For our structured material at 300 K, the strong magnetization relative to the unstructured material could be due to the mesostructure causing distortions of the crystal boundaries or asymmetric covalent Fe–O bonding, which have been shown to affect α-Fe_2_O_3_ magnetic properties.^37^

Furthermore, recent work simulating magnetic nickel with single gyroid morphology was able to support an assumption that spins in a strut of the gyroid would work together like a single macrospin that is aligned with the strut direction.^39^ While a single gyroid has does not have the same symmetry as the double gyroid studied here, this assumption may hold for a double gyroid morphology as well. It is worth noting that soft matter self-assembly derived structures like the double gyroid always have defects and grain boundaries (*e.g.*, see Fig. 3b and Fig. S3, ESI†). In the context of macrospins following the direction of gyroid struts, these defects and grain boundaries in the material could also have contributed to local magnetic anisotropy resulting in uncompensated spins, coercivity, and the asymmetry of the hysteresis from the structured sample *M–H* curves at 10 K. From literature, we do not expect any particular packing from iron oxide nanoparticles in an aluminosilicate matrix, rather the particles are expected to be dispersed throughout the matrix.^16^ However, this dispersion of nanoparticles throughout the struts of the matrix phase of the network gyroid structure may also contribute to differences in local magnetic anisotropy energies, due to differences in surface and core spins of the embedded nanoparticles.^33^ As expected at this early state, our work poses a number of questions that further in-depth studies need to address. It is clear from our results, however, that the nanoconfinement of the iron oxide phase induced by the gyroidal aluminosilicate nanostructure affects magnetic properties at 300 K and 10 K.

## 4. Conclusions

In this work, a triblock terpolymer-inorganic hybrid material with co-continuous morphology was developed and subsequently calcined to produce a mesoporous inorganic aluminosilicate with embedded ferromagnetic α-Fe_2_O_3_ nanoparticles in its walls. Structural analysis of this materials *via* SAXS and SEM is consistent with a double gyroid structure. To the best of our knowledge, this is the first example of a WF Fe_2_O_3_ material with mesoporous gyroid morphology. To structure-direct these inorganic materials, an ISO triblock terpolymer was synthesized and utilized. In solution, the ISO was mixed with iron(iii) ethoxide and a hydrolytic aluminosilicate sol; this solution was then cast into a PTFE dish, allowed to co-assemble during EISA, and subsequently calcined. The resulting films were then investigated with SAXS, SEM, and nitrogen physisorption, which, together, suggested that the calcined mesoporous materials had a co-continuous cubic double gyroid morphology with high-surface area and an average pore size of around 23 nm. SQUID vibrating-sample magnetometry *M*–*H* measurements showed that these porous materials had magnetic hysteresis at both 300 K and 10 K. Comparison between *M*–*H* and ZFC/FC curves of the mesostructured material and a chemically identical unstructured bulk material showed that the mesostructure did affect and enhance magnetic properties such as remnant magnetic field at zero applied magnetic field and magnetization magnitude at 300 K and 10 K. In particular, *M*–*H* curves at 10 K showed strong hysteresis, reminiscent of a ferromagnetic interaction, indicating an antiferromagnet that was not compensated; the hysteresis was also interestingly asymmetric for the structured inorganic at 10 K. We expect that the solution-based BCP SA route to mesoporous magnetic nanomaterials with co-continuous double gyroid morphology described here will open up pathways to a plethora of advanced magnetic nanomaterials, *e.g.*, through backfilling of the interconnected pores with a second (magnetic or superconducting) material, with anticipated impact on both fundamental science and technology.

## Author contributions

A. J. D.: investigation, formal analysis, visualization, writing – original draft, writing – review & editing. W. R. T. T. investigation, formal analysis, visualization, writing – original draft, writing – review & editing. W. M.: investigation. F. Y.: formal analysis and visualization. Y. L.: investigation. J. G. W.: investigation, writing – review & editing. R. B. V. D.: writing – review & editing. U. B. W.: funding acquisition, conceptualization, supervision, data analysis and interpretation, writing – original draft, writing – review & editing.

## Conflicts of interest

There are no conflicts of interest to declare.

## Supplementary Material

SM-020-D3SM01622F-s001
