# Supplementary material for: Block copolymer self-assembly derived mesoporous magnetic materials with three-dimensionally (3D) co-continuous gyroid nanostructure
Source: Soft Matter. 2024 Mar 5;20(12):2767–76. doi: 10.1039/d3sm01622f (PMC11650362; doi:10.1039/d3sm01622f)
Supplement: SM-020-D3SM01622F-s001 [file SM-020-D3SM01622F-s001.pdf]

Electronic Supplementary Material (ESI) for Soft Matter.
This journal is © The Royal Society of Chemistry 2024

Block copolymer self-assembly derived mesoporous magnetic materials with three-dimensionally (3D) co-continuous gyroid nanostructure

Amaury Jousset Drouhin, William R. T. Tait, William Moore, Fei Yu, Yuanzhi Lee, Joerg Werner, R. Bruce van Dover, Ulrich B. Wiesner

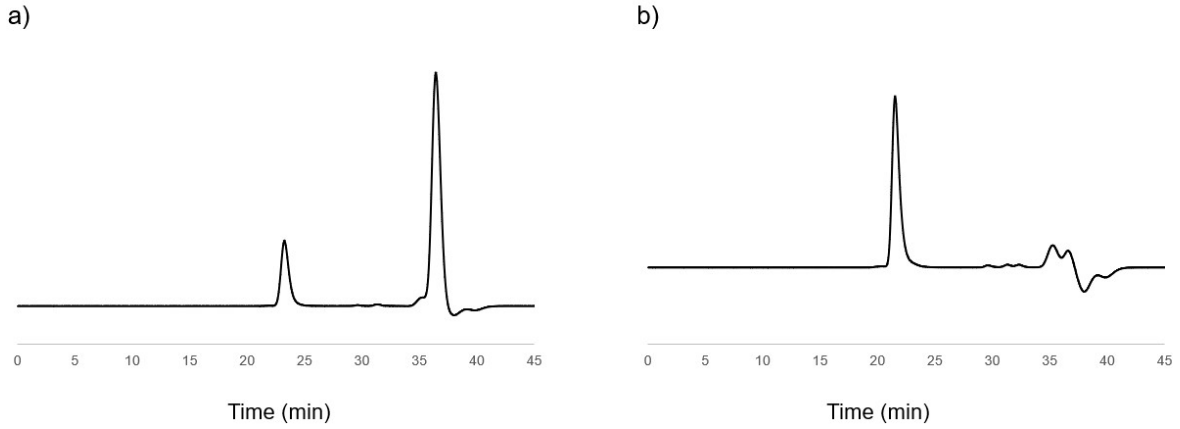

Figure S1: GPC of polyisoprene (PI) aliquot (a) and poly(isoprene-*block*-styrene) aliquot (b).

Table S1:

Sample	Mn (g/mol)	PDI
PI	10690 (by PI standard)	1.04
PI-*b*-PS	35329 (by PS standard)	1.08

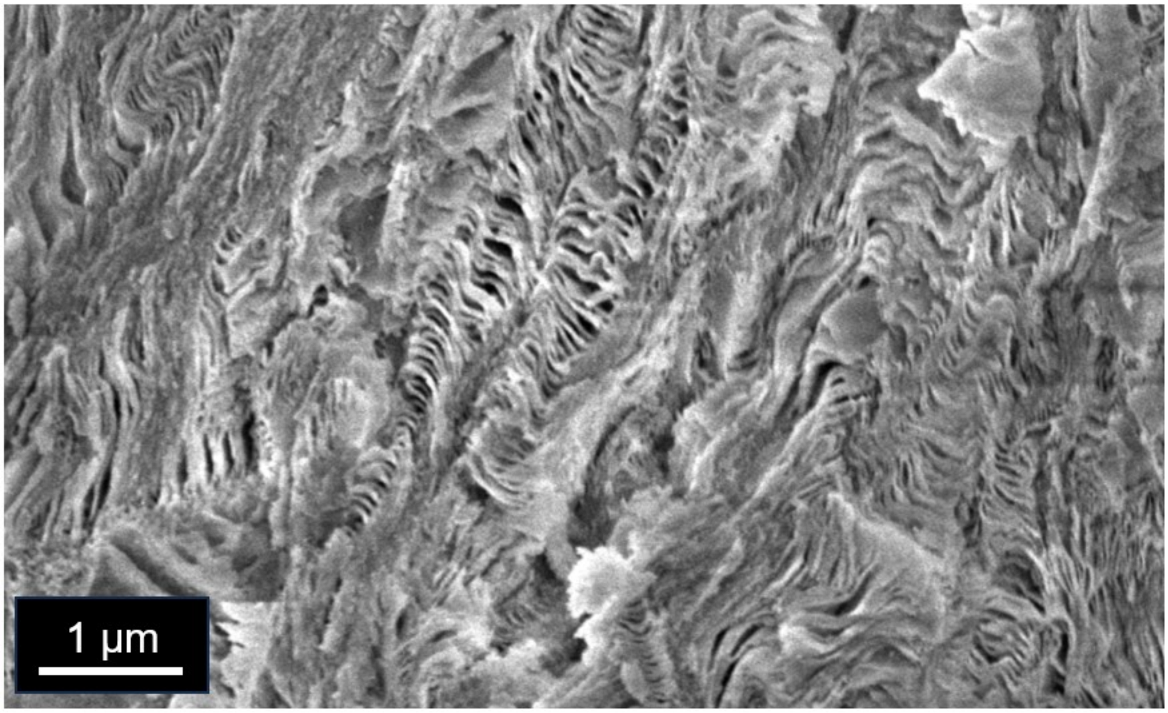

Figure S2: SEM images showing disordered material resulting from an inorganic additive with a molar ratio of 50:50 aluminosilicate precursor to iron(III) ethoxide.

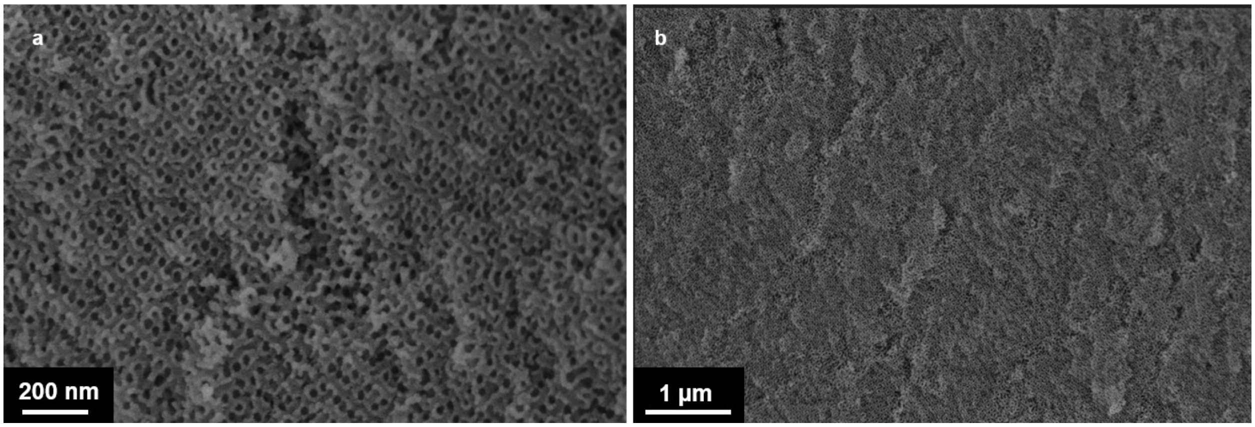

Figure S3: SEM images showing the final mesoporous material at two different magnifications.

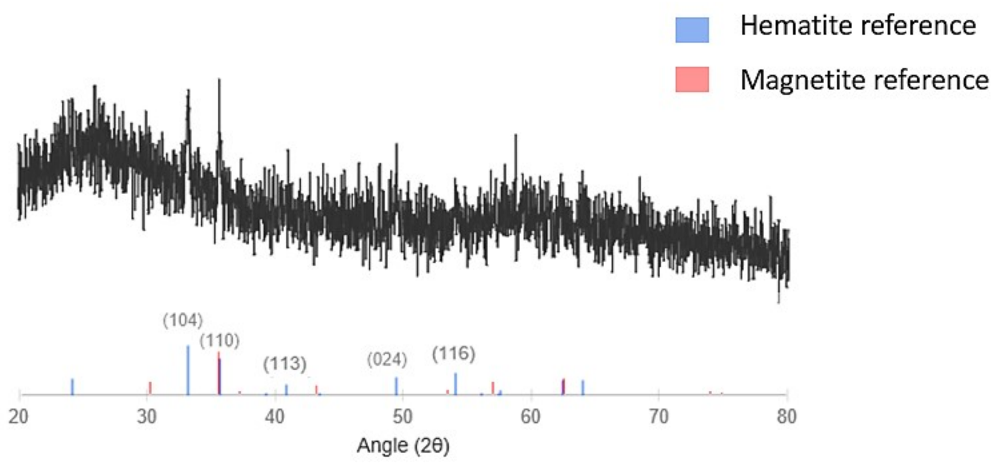

Figure S4: XRD pattern of unstructured sample with reference peaks for hematite (α-Fe_2O_3) and magnetite (Fe_3O_4) shown at the bottom from (PDF) #01-087-1164 [1] and (PDF) #01-079-0417,[2] respectively.

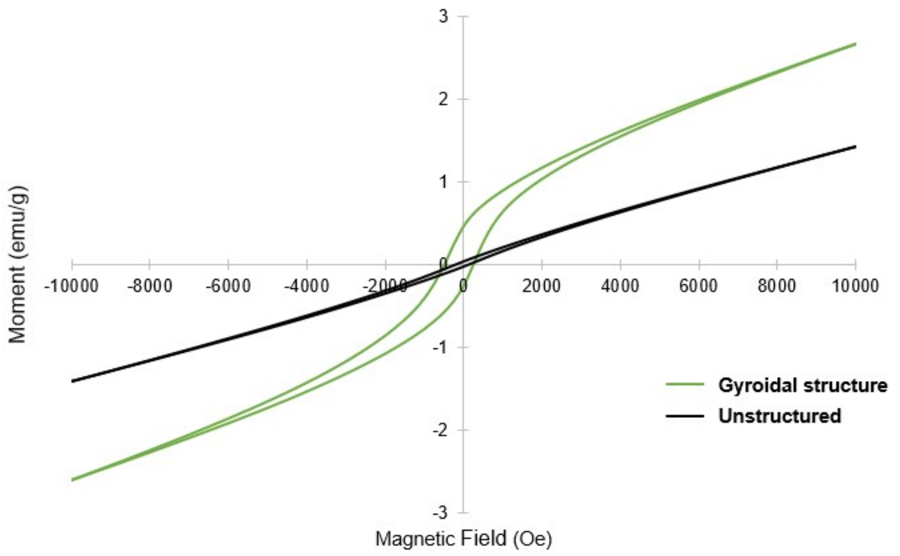

Figure S5: Comparison of M-H curves at 10 K for the BCP-derived mesoporous gyroidal (green) and unstructured bulk aluminosilicate/α-Fe_2O_3 inorganic (black) materials.

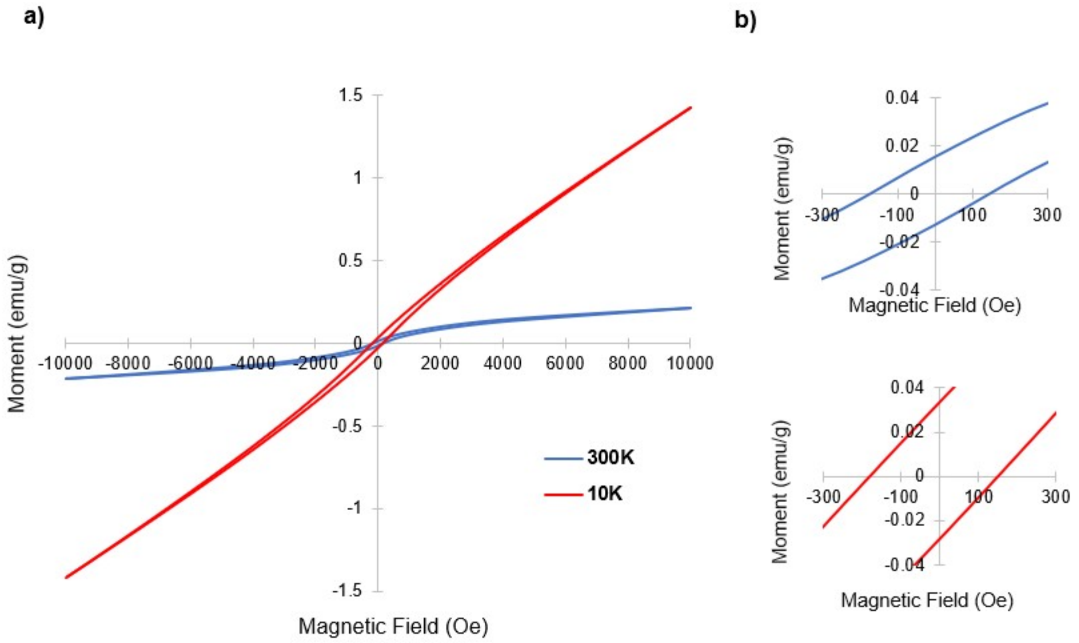

Figure S6:M-H curves at 300 K and 10 K obtained on the unstructured inorganic material (a). Zoomed-in versions showing the hysteresis near H=0 for 300 K (top) and 10 K (bottom) (b).

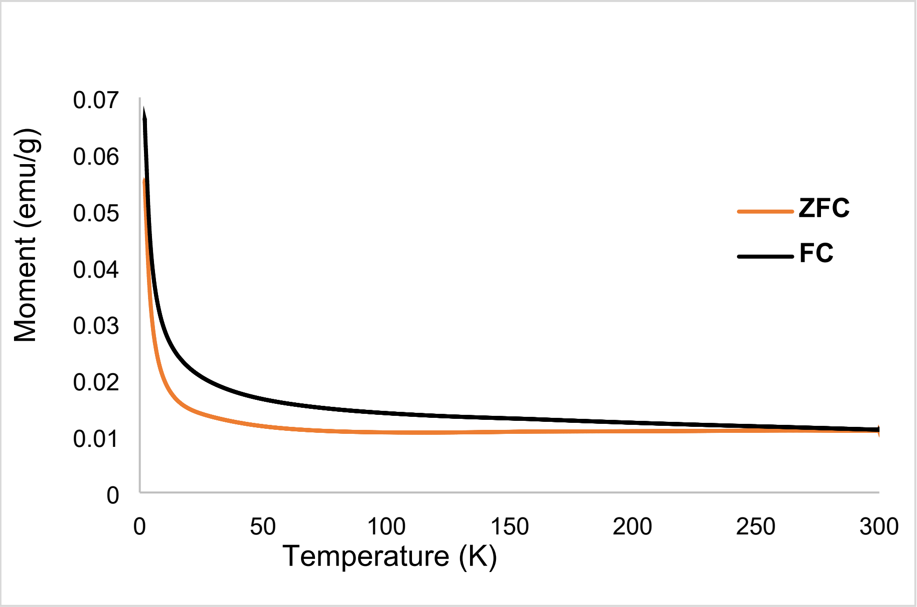

Figure S7: ZFC and FC curves for the unstructured inorganic material with expanded y-axis.

References:

1. Sawada, H. *Mater. Res. Bull.* 1996. 31, 141.
2. Fleet, M. E. *J. Solid State Chem.* 1986. 62, 75.
